# Mechanistic Evaluation of Panretinal Photocoagulation Versus Aflibercept in Proliferative Diabetic Retinopathy: CLARITY Substudy

**DOI:** 10.1167/iovs.17-23509

**Published:** 2018-08

**Authors:** Luke Nicholson, Roxanne Crosby-Nwaobi, Joana C. Vasconcelos, A. Toby Prevost, Jayashree Ramu, Amy Riddell, James W. Bainbridge, Philip G. Hykin, Sobha Sivaprasad

**Affiliations:** 1 National Institute for Health Research Moorfields Biomedical Research Centre, Moorfields Eye Hospital and University College London Institute of Ophthalmology, London, United Kingdom; 2 Imperial Clinical Trials Unit, School of Public Health, Imperial College London, London, United Kingdom; 3 King's Clinical Trials Unit at King's Health Partners, King's College London, London, United Kingdom

**Keywords:** aflibercept, diabetic retinopathy, oximetry, panretinal photocoagulation, retinal nonperfusion

## Abstract

**Purpose:**

The purpose of this study was to study the effects of panretinal photocoagulation (PRP) and intravitreal aflibercept on retinal vessel oxygen saturations, area of retinal nonperfusion, and area of neovascularization in proliferative diabetic retinopathy.

**Methods:**

This is a prospective randomized single center study. Forty patients with proliferative diabetic retinopathy were randomized to PRP or intravitreal aflibercept treatment for 52 weeks. Retinal oximetry and ultra-widefield angiography were performed at baseline and week 52. Ultra-widefield color fundus imaging was performed at baseline, week 12, and week 52. The outcomes were retinal arterio-venous oximetry differences (AVD), area of retinal nonperfusion, and area of neovascularization in disc areas (DA).

**Results:**

The AVD in the PRP group increased from 36.7% at baseline to 39.7%, whereas it decreased from 33.4% to 32.5% in the aflibercept group. The difference in AVD between groups at week 52 was 4.0% (95% confidence interval, −0.08, 8.8; *P* = 0.10). The baseline mean area of retinal nonperfusion of 125.1 DA and 131.2 DA in the PRP and aflibercept groups increased to 156.1 DA and 158.4 DA, respectively, at week 52 (*P* = 0.46). The median baseline area of neovascularization decreased from 0.98 DA to 0.68 DA in the PRP group and from 0.70 DA to 0 DA in the aflibercept group at week 12 (*P* = 0.019). At week 52, this measured 0.24 DA in the PRP group and 0 DA in the aflibercept group (*P* = 0.45).

**Conclusions:**

Intravitreal aflibercept achieved an earlier and complete regression of neovascularization in proliferative diabetic retinopathy compared with PRP. There were no significant differences in global change in intravascular oxygen saturation or areas of retinal nonperfusion between the two groups by 52 weeks.

Proliferative diabetic retinopathy (PDR) is characterized by the development of new vessels on the retina and/or optic disc. A key predictor of the development of these new vessels is the total area of retinal capillary nonperfusion. Ablation of areas of nonperfusion by panretinal photocoagulation (PRP) causes regression of new vessels identifying a relationship between nonperfusion and neovascularization.^1–4^ Since the 1970s, PRP has been the standard of care for patients with PDR. However, not all eyes with significant capillary nonperfusion develop new vessels and not all new vessels regress with PRP, indicating that other factors may be responsible for vessel regression.^5,6^

Both laboratory and clinical studies have proven that VEGF is a key angiogenic agent that stimulate retinal neovascularization.^7,8^ Injection of VEGF in vitreous of primates led to retinal neovascularization, and intravitreal anti-VEGF antibodies caused regression of these new vessels, demonstrating that intravitreal anti-VEGF agents cause vessel regression.^9,10^ Patients with PDR have high levels of VEGF in the vitreous that reduce with anti-VEGF injections.^8,11^

Currently, there are three anti-VEGF agents used in clinical practice. Bevacizumab and ranibizumab are humanized monoclonal antibodies that specifically bind to all isomers of VEGF-A, with ranibizumab being a Fab antibody fragment compared with a whole antibody, bevacizumab. A more recently introduced anti-VEGF agent is aflibercept, which blocks VEGF A, VEGF B, placental growth factor (PIGF), and Galectin-1.^12,13^ There are several clinical studies that have shown that short-term anti-VEGF therapy cause regression of new vessels in patients with PDR with reactivation of new vessels once anti-VEGF treatment is withdrawn.^10^ The Diabetic Retinopathy Clinical Research Network (DRCRnet) Protocol S study was the first study to show that monthly ranibizumab injections for at least 3 months followed by aggressive retreatment of recurrent or reactivated retinal neovascularization over 2 years resulted in noninferior visual outcomes compared with PRP with less need for vitrectomy and lower prevalence of diabetic macular edema.^14^ Eyes with PDR with no macular edema required a mean of seven injections in the first year and a further three injections in the second year, suggesting an anti-VEGF–related disease modulation over time. The CLARITY study that compared “pro re nata” intravitreal aflibercept treatment following a loading phase versus PRP in PDR patients without macular edema showed superior visual acuity outcomes, a higher proportion of new vessel regression, and lower incident vitreous hemorrhage and macular edema with aflibercept by 52 weeks.^5^ The patients required a loading phase of three injections followed by a median of one injection over the next 40 weeks.

These observations with anti-VEGF therapy raise a few questions. In particular, the exact mechanisms by which these new vessels regress rapidly with anti-VEGF therapy and the tendency to recur on withdrawal of anti-VEGF therapy in some eyes in the short-term remains unclear. Second, repeated anti-VEGF therapy resulted in fewer recurrences of neovascularization and less need for anti-VEGF therapy over time. This raises the question as to whether capillary nonperfusion stabilizes or improves with anti-VEGF therapy and whether this stabilization or improvement in capillary nonperfusion influences the observed course of new vessel regression. Retinal nonperfusion due to retinal vascular damage results in hypoxia and the mechanism of action of PRP is presumably by improving retinal oxygenation as evidenced by the narrowing of retinal vessel caliber and new vessel regression. Retinal hypoxia is also a major stimulus for VEGF, and the role of anti-VEGF on alleviating tissue hypoxia in proliferative diabetic retinopathy remains unclear. Studies on vitreous oxygen tension in PDR have shown significantly lower vitreous oxygen levels in eyes with PDR.^15^ However, measurement of vitreous oxygen levels to understand the effect of anti-VEGF therapy in PDR is invasive. Therefore, a measure of retinal intravascular oxygen saturations may indirectly provide information about the perfusion status of the retina. Recently, advances in spectral imaging have enabled noninvasive measurements of retinal intravascular oxygenation and vessel caliber with excellent reliability.^16,17^

The aim of this study was to test the hypothesis that repeated intravitreal aflibercept retards the progression of PDR by 52 weeks by improving retinal oxygen saturation. As direct measurement of tissue hypoxia is not feasible, we compared the change in retinal intravascular oxygen saturations, area of retinal nonperfusion, and area of retinal neovascularization in a mechanistic substudy of a randomized controlled trial that compared panretinal photocoagulation versus intravitreal aflibercept in proliferative diabetic retinopathy (CLARITY study).

## Methods

### Study Design

The CLARITY study is a multicenter, prospective, two-arm, parallel-group, randomized, noninferiority clinical trial that compared the outcomes of PRP versus repeated intravitreal aflibercept for proliferative diabetic retinopathy at 52 weeks. The study was granted approval by the National Research Ethics Committee Service London–South East (14/LO/0203). This mechanistic substudy was performed only at the National Institute for Health Research Biomedical Research Centre at Moorfields Eye Hospital, London, United Kingdom. Trial registration is ISRCTN32207582. The study was conducted according to the tenets of the Declaration of Helsinki, and informed consent was obtained from all patients prior to enrollment into the study.

### CLARITY Protocol Synopsis

The CLARITY study consisted of the main study on 232 patients with PDR and a substudy on the mechanistic evaluation in 40 patients. The main study protocol and primary outcome have been reported.^5^ In brief, adults with treatment naïve or post–laser-treated PDR with no macular edema were recruited from 22 United Kingdom ophthalmic centers and randomly assigned (1:1), with stratification including within center, to repeated intravitreal aflibercept or PRP standard care for 52 weeks using a web-based computer-generated system. Only one eye per patient was included in the study. In the PRP arm, patients were treated with initial PRP at 2-week intervals until completion of PRP and then reviewed every 8 weeks and retreated with supplemental PRP based on same retreatment criteria. In the aflibercept arm, patients were treated with a loading phase of intravitreal aflibercept injections at 4-week intervals and then assessed monthly and retreated with intravitreal aflibercept on a pro re nata basis based on prespecified retreatment criteria based on retinal vessel regression patterns for 52 weeks. Retinal color photographs were taken at all visits, and fundus fluorescein angiography was performed at baseline and week 52.

### Study Population

The mechanistic substudy was done on 40 eyes from 40 patients with proliferative diabetic retinopathy recruited for the CLARITY study at Moorfields Eye Hospital, London, between August 22, 2014, and November 30, 2015. These patients also underwent retinal oximetry and ultra-widefield color fundus imaging at baseline and 12 and 52 weeks. Ultra-widefield angiography was performed at baseline and week 52. Twenty patients were recruited into the aflibercept arm and 20 into the PRP arm.

### Retinal Oximetry

Retinal oximetry was performed using the retinal oximeter (Oxymap T1 device connected to Topcon TRC50-DX fundus camera; Oxymap ehf., Reykjavik, Iceland). It consists of a fundus camera with an attached image splitter, as well as a digital camera. The device captures images at two wavelengths, one sensitive to oxyhemoglobin (600 nm) and one isosbestic (570 nm), where the absorption spectra of oxyhemoglobin and hemoglobin cross. Computer software detects retinal vessels and uses relative light intensities inside and outside a vessel to calculate the optical density (light absorbance) of a vessel at both wavelengths. The hemoglobin oxygen saturation (SO_2_) of a vessel can, therefore, be calculated because the optical density ratio at these wavelengths has been shown to have an approximately inverse linear relationship with SO_2_. Optic disc–centered images were captured through dilated pupils, and the images were 1200 × 1600 pixels and covered a 50° field of central retina. The images were captured for both eyes, but only the study eye is used in this analysis. Images were analyzed using the Oxymap Analyzer software (Oxymap ehf.). A minimum vessel width of eight pixels was set, and vessels less than eight pixels were automatically excluded from the analysis. Oximetry measurements were performed using previously reported methods whereby the intrasubject reproducibility had an intraclass correlation coefficient of 0.98.^17^ In brief, an initial central circle was used to delineate the optic disc. Two additional measurement circles are made, one three times the diameter of the central circle and the following circle 1.5 times the diameter of the central circle. The area between these two additional circles centered on the optic disc was analyzed. Areas where vessel detection would prove inaccurate (branching, overlapping, intersecting vessels, background hemorrhage, or underlying laser scars), and segments of vessels less than 50 pixels in length were excluded. Vessels to be measured were selected manually. Values for each selected vessel were obtained and subsequently averaged. The retinal oximetry data streams were extracted from the source data file. Data collected from the Oxymap software included the mean arteriolar oxygen saturations (SaO_2_), mean venular oxygen saturations (SvO_2_), mean arterial vessel diameter, and mean venular diameter.

### Ultra-Widefield Color Fundus Photograph

All 40 patients underwent ultra-widefield color fundus photography using the Optos 200TX (Optos Plc, Dumfermline, Scotland) ultra-widefield system. The macular-centered 200° images were used for analysis. Each central color fundus image was superimposed with the concentric rings template.^18^ In brief, this validated method incorporates a macular ring with a radius of 2.5 disc diameters (DD) and five additional concentric rings (rings 1–5), each with a 2.5 DD increment in radius. Each of the six rings (ring M and 1–5) were divided into 12 segments. The area of each cell in each concentric ring was modified based on the enlargement factor identified using three-dimensional printed model eyes.^19^ The enlargement factor for rings M, 1, 2, 3, 4, and 5 were 1.08, 1.20, 1.34, 1.54, 1.81, and 1.97, respectively. Ultra-widefield fluorescein angiogram and ultra-widefield red free images were used as a reference in identifying the new vessels. The areas of new vessels on ultra-widefield color fundus images were measured using ImageJ (National Institutes of Health, Bethesda, MD, USA) in pixels and then converted to disc areas based on the pixel count for the corresponding disc area for each image. The areas of new vessels were corrected based on the location using the enlargement factors for each ring. The presence of disc new vessels and/or new vessels elsewhere was also recorded.

### Ultra-Widefield Fundus Fluorescein Angiography

Ultra-widefield fundus fluorescein angiography was performed using the Optos 200TX ultra-widefield system. The fluorescein angiography images were acquired after intravenous bolus infusion of 5 mL 20% fluorescein sodium. The images were acquired at transit phase (up to 45 seconds), arteriovenous phases (3 to 4 minutes), and late frames at 5 to 7 minutes. A single investigator (LN) identified the best macula-centered fluorescein angiography (FA) image in the arteriovenous phase from the FA series of each eligible eye. Images where clear delineation between perfused and nonperfused retina could not be made were excluded from the analysis. A correction factor was applied for the flattening of the three-dimensional image to a two-dimensional image using the Optos V2 Vantage Pro software. Each selected image was superimposed with the concentric rings template. The previously validated concentric rings method was used to measure retinal nonperfusion. In brief, each segment is graded as ungradable, nonperfused, or perfused if 50% or more of the segment is involved. In addition to quantifying nonperfusion, the concentric rings method allows documentation of location of nonperfusion. The area of each cell in each concentric ring was modified based on the enlargement factor identified using the three-dimensional printed model eyes as described previously. Ring 5 was excluded from our analysis as the number of ungradable cells has been reported to be high and suitable for steered images only. Lasered areas were scored based on the area in between laser scars and the overall assessment of the perfusion in the assessed segment. Where ungradable, this was scored as ungradable. Measurements were performed for all baseline images for both PRP and aflibercept eyes in one session, and the exit images were analyzed for both PRP and aflibercept at a separate time period to reduce any potential bias. Assessors were masked to the treatment arm.

### Study Outcomes

The primary outcome of this study was to compare the change in arterio-venous differences in retinal vessel oxygenation at week 12 and week 52 compared with baseline for the eyes treated with panretinal photocoagulation and intravitreal aflibercept. The secondary outcomes were to compare the change in area of retinal nonperfusion at week 52 compared with baseline and the change in area of neovascularization at week 12 and week 52 with baseline for eyes treated with panretinal photocoagulation and intravitreal aflibercept.

### Statistical Analysis

The oximetry measurements and area of retinal nonperfusion were described using means, SD or SE, or medians and interquartile range (IQR).The outcomes were compared between both aflibercept and PRP treatment arms at both 12 and 52 weeks for oximetry and area of retinal nonperfusion at 52 weeks using analysis of covariance (ANCOVA) adjusting for the baseline of the outcome under analysis.^20^ Comparison between the area of new vessels was performed using a Mann-Whitney test because the distribution was skewed. Statistical significance was set at 0.05, although 95% confidence intervals (CIs) are presented and interpreted because this is a substudy within a trial.

## Results

**Figure 1:**
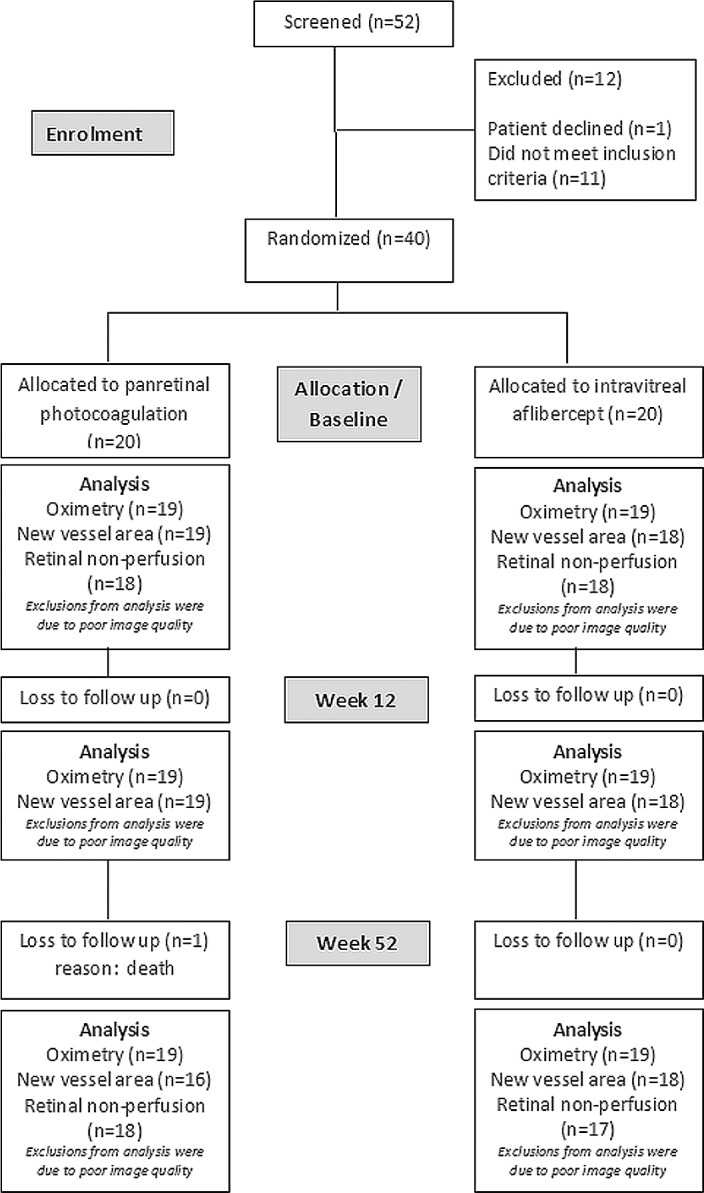
Participant flow diagram from screening, baseline, week 12, and week 52 with number of eyes analyzed for retinal oximetry, area of new vessels, and area of retinal nonperfusion at each time points.

**Figure 2:**
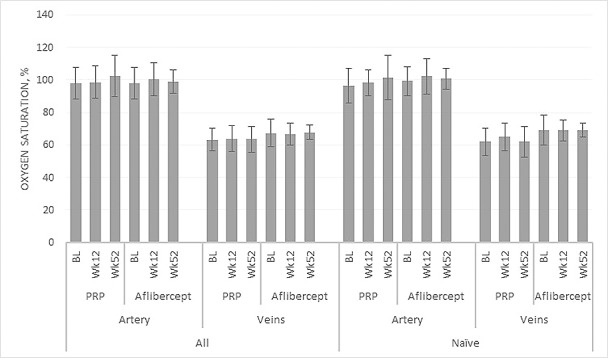
Retinal oximetry measurements for retinal arteries and veins for eyes treated with PRP and intravitreal aflibercept at baseline, week 12, and week 52 for all eyes and the treatment-naïve subgroup.

The mean age of the cohort was 46.7 years (SD, 12.7), and 32.5% were females. The participant flow diagram is presented in Figure 1. Retinal oximetry measurements revealed fairly similar arterial and venous saturations in both treatment groups at week 12 and week 52 (Fig. 2). The mean arterio-venous oxygen saturation difference (AVD) at baseline was 36.7% (SD, 10.2%) in the PRP group and 33.4% (SD, 8.2%) in the aflibercept group. At week 12, the AVD was 36.1% and 35.5% for the PRP and aflibercept groups, respectively. At week 52, the AVD increased in the PRP arm to 39.7% and minimally changed in the aflibercept arm to 32.5%. The effect size of the difference between the two groups at 52 weeks, adjusted for baseline, was 4.0% (95% CI, 0.08, 8.8); Mean arterial and venous diameter decreased in both groups at week 52, again with no significant difference between groups (Supplementary Material).

The mean total area of retinal nonperfusion increased in both PRP and aflibercept groups at 52 weeks predominantly in the outer zones, with a mean adjusted difference of 12.0 DA (95% CI, −20.7, 44.7) for total area of nonperfusion between the two groups. At baseline, the total area of retinal nonperfusion in the PRP group was 125.1 DA and 131.2 DA in the aflibercept group. At week 52, this was 156.1 DA and 158.4 DA in the PRP and aflibercept groups, respectively. Focusing on posterior pole nonperfusion alone, the mean area of nonperfusion at baseline was 15.4 DA and 16.9 DA for the PRP and aflibercept groups, respectively. This was measured as 19.6 DA and 14.8 DA for the PRP and aflibercept groups at week 52, respectively. The difference between the change from baseline between the PRP and aflibercept was less in the aflibercept group, with an adjusted difference of −5.0 DA (95% CI, −13.4, 3.5; *P* = 0.24). This is detailed in Table 1.

**Table 1:**
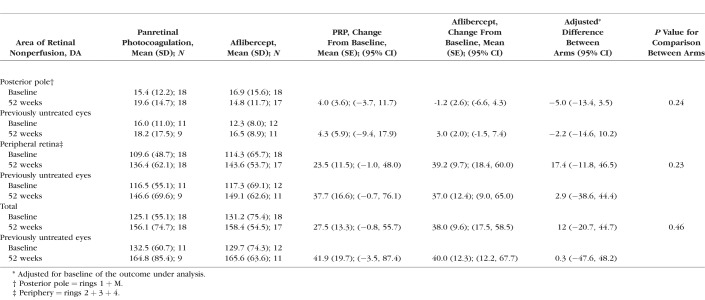
Area of Retinal Nonperfusion in Disc Areas and Change From Baseline Based on Total Area, Posterior Pole, and Periphery for All Eyes and Treatment-Naïve Eyes Treated With Intravitreal Aflibercept and PRP Over 1 Year

**Figure 3:**
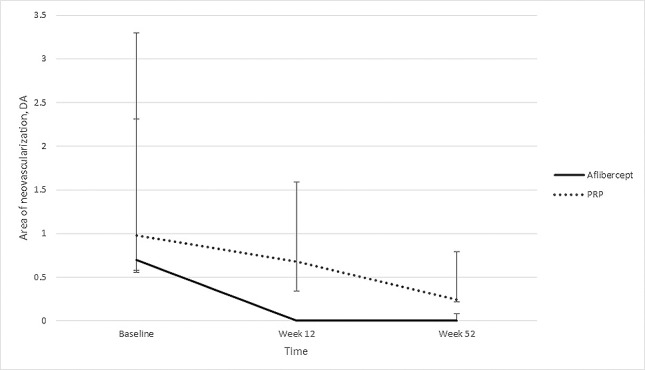
Area of neovascularization between eyes treated with intravitreal aflibercept and PRP at baseline, week 12, and week 52 reported in median and error bars describing the IQR.

The median total area of retinal neovascularization was 0.98 DA (IQR, 0.42, 1.33) in the PRP group and 0.70 DA (IQR, 0.12, 2.6) in the aflibercept group at baseline. At week 12, there was a complete regression for the aflibercept group, whereas the median total area of new vessels for the PRP group was 0.68 DA (*P* = 0.019). The median total area of neovascularization was not statistically significant between groups at week 52: 0.24 DA in the PRP group and 0 DA in the aflibercept group (*P* = 0.45). This is elaborated in Figure 3. All eyes experienced total regression of new vessels in the aflibercept group at week 12, whereas in the PRP group, 78.9% (15 of 19) showed partial regression, with no eyes experiencing total regression; 21.1% (4 of 19) of eyes in the PRP group had no regression at week 12. At the end of the study, 25% (4 of 16) of eyes in the PRP group had total regression of new vessels, 62.5% (10 of 16) had partial regression, and 12.5% (2 of 16) had no regression compared with baseline. As for the aflibercept group, 55.6% (10 of 18) had total regression and 44.4% (8 of 18) had partial regression. This is represented in Table 2.

**Table 2:**
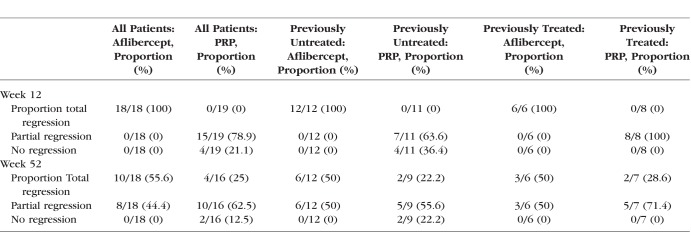
Regression Status at 12 and 52 Weeks in Aflibercept and PRP Arms and Within Previous Untreated and Previously Treated Eyes

## Discussion

The results from this mechanistic substudy tested within the CLARITY study, a prospective randomized clinical trial comparing aflibercept and PRP for proliferative diabetic retinopathy, suggest that, despite significant rapid regression of retinal neovascularization with aflibercept compared with PRP, the changes in total area of retinal capillary nonperfusion and intravascular oxygen saturation at week 52 compared with baseline between the aflibercept and PRP cohorts are not significant.

This study corroborated several other reports that has shown rapid regression of retinal neovascularization or significant improvement in diabetic retinopathy severity with anti-VEGF therapy.^5,10,14,21–24^ The new vessels regressed completely after the three loading injections of aflibercept and more than half of eyes (10 of 18) remained completely regressed at 52 weeks despite an average of 1.4 supplemental aflibercept injections over the next 40 weeks, suggesting an immediate but prolonged effect of aflibercept on regression of new vessels. On the contrary, the new vessel regression was much slower and incomplete with PRP by 52 weeks. The reason for rapid regression of new vessels with anti-VEGF therapy is unclear. We evaluated whether improved tissue oxygenation with anti-VEGF therapy contribute to new vessel regression. As measuring tissue oxygen levels is invasive, measuring global intravascular oxygen saturation around the optic disc noninvasively by retinal oximetry is the nearest surrogate marker of tissue hypoxia.^25^ A larger difference between the arterio-venous oxygen saturation represents better retinal tissue perfusion by the intervening capillaries. In contrast, minimal or small differences between the oxygen saturation in the arteries and veins indicate shunting of blood from arteries to vein bypassing the tissue due to capillary nonperfusion.

In this study, we observed very minimal change in arterio-venous oxygen saturation after aflibercept therapy at 52 weeks compared with an increase in the PRP group, resulting in a 4% (95% CI, −0.08, 8.8) difference between groups. The effect size of this change that relates to a clinically meaningful impact on retinal tissue hypoxia is unknown. However, the results concur with the fact that PRP tends to address the hypoxic insult by ablating the high oxygen consuming photoreceptors in the outer retina and improving oxygenation of the inner retina from the choroid.^26,27^ On the contrary, anti-VEGF therapy did not result in a demonstrable change in intravascular oxygen saturation by 52 weeks, suggesting that the disease modulation induced by anti-VEGF at least in the short term is not caused by direct and immediate improvement in oxygenation. Diabetic retinopathy is a slowly progressing disease. Any change in tissue hypoxia may take time to manifest as a change in intravascular oxygen saturation. This is substantiated by the fact that a difference in intravascular oxygen saturation in the PRP arm was only observed at 52 weeks and not at 12 weeks, despite the dramatic ablation of the retina. Based on our results, we believe that we require a larger sample size with a longer follow-up to better identify any small differences in intravascular oxygenation between treatment groups. This is why we have interpreted the 95% CI to distinguish evidence of absence of effect conclusions from absence of evidence effect conclusions.^28^ Currently, we are also not aware of the amount of change in tissue hypoxia that is required to reflect a change in intravascular oxygen saturations. Further studies in this area are required to better understand this relation.

Campachiaro et al. postulated that VEGF driven leucostasis may in fact cause retinal capillary closure and worsen retinal ischemia, and anti-VEGF therapy may act by inhibiting this positive feedback loop.^29,30^ Our study shows that the effect of inhibiting this positive feedback loop on tissue oxygenation by anti-VEGF may indeed be slow. This effect is independent to the total regression of new vessels, which is an immediate effect. On the contrary, the effect of PRP may be a direct mechanical effect of improving retinal oxygenation from the choroid rather than by inhibiting the VEGF drive per se as evidenced by the lack of change in vitreous VEGF levels soon after PRP. The VEGF level is reported to reduce in quiescent PDR, suggesting the secondary effects of PRP on VEGF. ^8,31^

We also found no change in total area of retinal nonperfusion between treatment groups by 52 weeks after excluding a 45-point or higher difference between arms. There have been reports of possible reversal or stabilization of retinal nonperfusion following anti-VEGF and intravitreal steroid therapy identifying VEGF as a key factor contributing to retinal nonperfusion.^28,31,32^ These studies used different methods in calculating retinal nonperfusion. Campochiaro et al. evaluated nonperfusion in the posterior pole only and this may in fact be a more reliable estimate of risk of retinal neovascularization than utilizing the whole of the retinal gradable area on wide-field imaging.^29^ The positive feedback loop is well illustrated in the analysis of posterior retinal nonperfusion in the RISE and RIDE study that showed a decrease in the number of patients with no posterior nonperfusion by 24 months that then stabilized with ranibizumab therapy by 36 months.^30^

Multiple reasons may explain why we found no difference in retinal nonperfusion between treatment groups. First, we included eyes with active neovascularization following initial PRP in both arms. The amount of positive feedback loop and resultant retinal nonperfusion after initial PRP may indeed behave differently with further treatment compared with treatment naïve eyes.

Second, due to the various phenotypes of retinal nonperfusion in diabetes, an ischemic index or a concentric ring method of calculating global retinal nonperfusion may not reliably correlate with new vessel formation. This is further substantiated by the wide CI noted in our study that is wide enough to support a 20-point effect in the other direction (95% CI, −20.7, 44.7). This variable progression of retinal nonperfusion in our cohort may also explain the varied response to PRP in clinical practice and the need for repeated laser treatments in some cases. Finally, isolating the change in area of nonperfusion to rings M and 1 that represents the posterior pole, we have found that, in the PRP group, there is an increase in the mean area of nonperfusion, but in the aflibercept group, this has decreased; the difference was found to be −5.0 DA (95% CI, −13.4, 3.5; *P* = 0.24). Acknowledging the importance of posterior pole nonperfusion having a larger effect in neovascularization, this could potentially provide an explanation to the lasting effects of aflibercept in suppressing neovascularization beyond its pharmacodynamics.^30,32^ This was only evident in all eyes and not seen in treatment naïve eyes, although the numbers for the treatment naïve eyes are small.

In our cohort of eyes with PRP, the baseline total area of retinal nonperfusion was 128.2 DA. In previous reports on ultra-widefield angiography in diabetic retinopathy, the baseline ischemic index was reported to be 24% to 31.9%, which roughly converts to 84 to 108 DA.^33,34^ These studies reported a cohort of eyes with diabetic retinopathy but not specifically eyes with proliferative retinopathy. Therefore, our findings of a higher baseline area of nonperfusion are expected but also raise the question as to whether there is a point of no: return beyond which anti-VEGF therapy may not stabilize or reverse retinal nonperfusion. In our study, both treatment groups showed a progression of retinal nonperfusion by 52 weeks. These eyes may indeed behave similar to ischemic central retinal vein where the progression to neovascular complications was not absolved with anti-VEGF therapy.^35^

The strengths of this study include the prospective and randomized study design with prespecified outcomes to study these particular factors. The methodology used for measuring retinal oximetry and retinal nonperfusion has also been validated and proven to be reliable. Furthermore, the findings of this study are novel and to our knowledge have not been reported previously. The limitation of this study is that, as it is an exploratory study addressing several factors, the sample size may allow type 2 errors, thereby accepting the null hypothesis, as the study may be underpowered for certain outcomes, as seen where 95% CIs include differences that are clinically significant. Furthermore, the measurement of retinal nonperfusion in eyes receiving PRP is a challenge, but we ensured that only images of good quality whereby clear delineation can be made between perfused and nonperfused retina are included in the analysis. This measurement error, if any, is applicable to both arms and should not influence the change between arms. There was also no significant difference in the number of ungradable segments in the PRP and aflibercept cohorts. Blinding of assessors is difficult in naïve eyes treated with aflibercept as the lack of laser scars will suggest the treatment arm.

In conclusion, we showed that the change in intravascular oxygen saturation observed at 52 weeks in the PRP arm corroborate with our understanding of the mechanism of PRP. In contrast, the anti-VEGF effect on retinal hypoxia may indeed be slow and minimal. On the contrary, eyes receiving intravitreal aflibercept achieve an earlier and more complete regression of new vessels compared with eyes receiving conventional PRP by 52 weeks and precede any changes in total area of capillary nonperfusion. Our study results highlight that these treatment options may have synergistic effects in the management of PDR. Further studies with a larger sample size of PDR with longer follow-up focusing on central retinal nonperfusion are required to ratify these observations.

## Supplementary Material

Supplement 1

## References

[1] Wand M, Dueker DK, Aiello LM, Grant WM (1978). Effects of panretinal photocoagulation on rubeosis iridis, angle neovascularization, and neovascular glaucoma. *Am J Ophthalmol*.

[2] Little HL (1985). Treatment of proliferative diabetic retinopathy. Long-term results of argon laser photocoagulation. *Ophthalmology*.

[3] The Diabetic Retinopathy Study Research Group (1978). Photocoagulation treatment of proliferative diabetic retinopathy: the second report of diabetic retinopathy study findings. *Ophthalmology*.

[4] Muqit MMK, Young LB, McKenzie R (2013). Pilot randomised clinical trial of Pascal TargETEd Retinal versus variable fluence PANretinal 20 ms laser in diabetic retinopathy: PETER PAN study. *Br J Ophthalmol*.

[5] Sivaprasad S, Prevost AT, Vasconcelos JC (2017). Clinical efficacy of intravitreal aflibercept versus panretinal photocoagulation for best corrected visual acuity in patients with proliferative diabetic retinopathy at 52 weeks (CLARITY): a multicentre, single-blinded, randomised, controlled, phase 2b, non-inferiority trial. *Lancet Lond Engl*.

[6] Zhou A-Y, Zhou C-J, Yao J, Quan Y-L, Ren B-C, Wang J-M (2016). Panretinal photocoagulation versus panretinal photocoagulation plus intravitreal bevacizumab for high-risk proliferative diabetic retinopathy. *Int J Ophthalmol*.

[7] Lebherz C, Maguire AM, Auricchio A (2005). Nonhuman primate models for diabetic ocular neovascularization using AAV2-mediated overexpression of vascular endothelial growth factor. *Diabetes*.

[8] Aiello LP, Avery RL, Arrigg PG (1994). Vascular endothelial growth factor in ocular fluid of patients with diabetic retinopathy and other retinal disorders. *N Engl J Med*.

[9] Ozaki H, Hayashi H, Vinores SA, Moromizato Y, Campochiaro PA, Oshima K (1997). Intravitreal sustained release of VEGF causes retinal neovascularization in rabbits and breakdown of the blood-retinal barrier in rabbits and primates. *Exp Eye Res*.

[10] Arevalo JF, Lasave AF, Wu L (2017). Intravitreal bevacizumab for proliferative diabetic retinopathy: results from the Pan-American Collaborative Retina Study Group (PACORES) at 24 months of follow-up. *Retina*.

[11] Forooghian F, Kertes PJ, Eng KT (2016). Alterations in intraocular cytokine levels following intravitreal ranibizumab. *Can J Ophthalmol*.

[12] Kanda A, Noda K, Saito W, Ishida S (2015). Aflibercept traps Galectin-1, an angiogenic factor associated with diabetic retinopathy. *Sci Rep*.

[13] Stewart MW (2013). Aflibercept (VEGF Trap-Eye) for the treatment of exudative age-related macular degeneration. *Expert Rev Clin Pharmacol*.

[14] Writing Committee for the Diabetic Retinopathy Clinical Research Network (2015). Panretinal photocoagulation vs intravitreous ranibizumab for proliferative diabetic retinopathy: a randomized clinical trial. *JAMA*.

[15] Lange CAK, Stavrakas P, Luhmann UFO (2011). Intraocular oxygen distribution in advanced proliferative diabetic retinopathy. *Am J Ophthalmol*.

[16] Yip W, Siantar R, Perera SA (2014). Reliability and determinants of retinal vessel oximetry measurements in healthy eyes. *Invest Ophthalmol Vis Sci*.

[17] Man REK, Kawasaki R, Wu Z (2013). Reliability and reproducibility of retinal oxygen saturation measurements using a predefined peri-papillary annulus. *Acta Ophthalmol (Copenh)*.

[18] Nicholson L, Vazquez-Alfageme C, Ramu J (2015). Validation of concentric rings method as a topographic measure of retinal nonperfusion in ultra-widefield fluorescein angiography. *Am J Ophthalmol*.

[19] Nicholson L, Vazquez-Alfageme C, Clemo M (2018). Quantifying retinal area in ultra-widefield imaging using a 3-dimensional (3-D) printed eye model. *Ophthalmol Retina*.

[20] Vickers AJ, Altman DG (2001). Statistics notes: analysing controlled trials with baseline and follow up measurements. *BMJ*.

[21] Nguyen QD, Brown DM, Marcus DM (2012). Ranibizumab for diabetic macular edema: results from 2 phase III randomized trials: RISE and RIDE. *Ophthalmology*.

[22] Ip MS, Domalpally A, Hopkins JJ, Wong P, Ehrlich JS (2012). Long-term effects of ranibizumab on diabetic retinopathy severity and progression. *Arch Ophthalmol*.

[23] Brown DM, Schmidt-Erfurth U, Do DV (2015). Intravitreal aflibercept for diabetic macular edema: 100-week results from the VISTA and VIVID studies. *Ophthalmology*.

[24] Bressler SB, Liu D, Glassman AR (2017). Change in diabetic retinopathy through 2 years: secondary analysis of a randomized clinical trial comparing aflibercept, bevacizumab, and ranibizumab. *JAMA Ophthalmol*.

[25] Stefánsson E, Olafsdottir OB, Einarsdottir AB (2017). Retinal oximetry discovers novel biomarkers in retinal and brain diseases. *Invest Ophthalmol Vis Sci*.

[26] Stefansson E (1990). Oxygen and diabetic eye disease. *Graefes Arch Clin Exp Ophthalmol*.

[27] Stefansson E, Landers MB, Wolbarsht ML (1981). Increased retinal oxygen supply following pan-retinal photocoagulation and vitrectomy and lensectomy. *Trans Am Ophthalmol Soc*.

[28] Altman DG, Bland JM (1996). Absence of evidence is not evidence of absence. *Aust Vet J*.

[29] Campochiaro PA, Bhisitkul RB, Shapiro H, Rubio RG (2013). Vascular endothelial growth factor promotes progressive retinal nonperfusion in patients with retinal vein occlusion. *Ophthalmology*.

[30] Campochiaro PA, Wykoff CC, Shapiro H, Rubio RG, Ehrlich JS (2014). Neutralization of vascular endothelial growth factor slows progression of retinal nonperfusion in patients with diabetic macular edema. *Ophthalmology*.

[31] Shimura M, Yasuda K, Nakazawa T (2009). Panretinal photocoagulation induces pro-inflammatory cytokines and macular thickening in high-risk proliferative diabetic retinopathy. *Graefes Arch Clin Exp Ophthalmol*.

[32] Nicholson L, Vazquez-Alfageme C, Patrao NV (2017). Retinal nonperfusion in the posterior pole is associated with increased risk of neovascularization in central retinal vein occlusion. *Am J Ophthalmol*.

[33] Querques L, Parravano M, Sacconi R, Rabiolo A, Bandello F, Querques G (2017). Ischemic index changes in diabetic retinopathy after intravitreal dexamethasone implant using ultra-widefield fluorescein angiography: a pilot study. *Acta Diabetol*.

[34] Sim DA, Keane PA, Rajendram R (2014). Patterns of peripheral retinal and central macula ischemia in diabetic retinopathy as evaluated by ultra-widefield fluorescein angiography. *Am J Ophthalmol*.

[35] Brown DM, Wykoff CC, Wong TP (2014). Ranibizumab in preproliferative (ischemic) central retinal vein occlusion: the rubeosis anti-VEGF (RAVE) trial. *Retina*.

