# Supplementary material for: Mechanistic Evaluation of Panretinal Photocoagulation Versus Aflibercept in Proliferative Diabetic Retinopathy: CLARITY Substudy
Source: Invest Ophthalmol Vis Sci. 2018 Aug;59(10):4277–84. doi: 10.1167/iovs.17-23509 (PMC6108778; doi:10.1167/iovs.17-23509)
Supplement: Supplement 1 [file iovs-59-10-30_s01.pdf]

Supplementary file 1: Retinal oximetry measurements for eyes treated with intravitreal Aflibercept and PRP and the change over 12 weeks and one year in measures of mean arterial and mean venous diameter, overall and within the previously untreated subgroups

**Area of retinal non-perfusion, disc areas**	**Panretinal photocoagulation, Mean (SD); N**	**Aflibercept, Mean (SD); N**	**Panretinal photocoagulation, Change from Baseline Mean (SE)**	**Aflibercept, Change from Baseline Mean (SE)**	**Adjusted difference between arms (95% CI)**	***p*-value**
**Mean arterial diameter**						
Baseline	113.5 (10.2); 19	111.8 (16.3); 19				
12-week	111.0 (8.5); 18	108.8 (11.0); 18	-3.3 (2.1)	-2.7 (3.6)	-1.3 (-7.3, 4.8)	0.67
52-week	107.4 (9.3); 18	109.3 (14.4); 18	-5.5 (2.1)	-3.4 (2.4)	2.0 (-3.8, 7.8)	0.48
Previously untreated						
Baseline	114.3 (12.2); 11	111.3 (17.6); 13				
12-week	112.3 (9.2); 11	108.8 (11.5); 12	-2.0 (3.0)	-2.0 (4.6)	-2.3 (-10.2, 5.7)	
52-week	108.4 (9.1); 10	109.7 (15.7); 13	-5.0 (2.9)	-1.6 (2.1)	2.8 (-3.6, 9.3)	
**Mean venous diameter**						
Baseline	143.4 (13.1); 19	146.7 (16.6); 19				
12-week	144.9 (14.8); 18	144.1 (12.3); 18	0.2 (2.6)	-3.3 (4.6)	-1.7 (-10.4, 7.0)	0.69
52-week	140.5 (12.7); 18	143.9 (15.4); 18	-3.8 (2.0)	-1.4 (3.8)	2.8 (-5.0, 10.7)	0.47
Previously untreated						
Baseline	146.4 (11.8); 11	142.6 (12.9); 13				
12-week	145.4 (12.4); 11	145.9 (13.6); 12	-1.0 (3.4)	2.4 (4.3)	1.9 (-8.5, 12.4)	
52-week	144.5 (8.4); 10	145.0 (15.7); 13	-3.8 (1.6)	2.3 (2.7)	5.4 (-1.9, 12.8)	
